# Prognostic value of marital status on stage at diagnosis in hepatocellular carcinoma

**DOI:** 10.1038/srep41695

**Published:** 2017-01-31

**Authors:** Wenjie Zhang, Xiaochen Wang, Ruyi Huang, Kangpeng Jin, Guangyan Zhangyuan, Weiwei Yu, Yin Yin, Hai Wang, Zekuan Xu, Beicheng Sun

**Affiliations:** 1 Liver Transplantation Center of the First Affiliated Hospital and State Key Laboratory of Reproductive Medicine, Nanjing Medical University, Nanjing, Jiangsu Province, P.R. China; 2 Department of General Surgery, The First Affiliated Hospital of Nanjing Medical University, Nanjing, Jiangsu Province, P.R. China

## Abstract

Marital status have been found as an independent prognostic factor for survival and spousal support could provide a survival advantage in various cancer types. However, the specific effect of marital status on survival in hepatocellular carcinoma (HCC) has not been explored in detail. In this study, we used the Surveillance, Epidemiology and End Results program to identify iagnosed with HCC between 1988 and 2007. Kaplan-Meier methods and multivariable Cox regression models were used to analyze long-term cancer-specific survival (CSS) outcomes and risk factors stratified by marital status. There were significant differences among these different marital status subgroups with regard to 5-year CSS rates (P < 0.001). Married HCC patients had a better 5 year CSS rate than those unmarried patients, and widowed patients were more likely to die of their cancer. A stratified analysis showed that widowed patients always had the lowest CSS rate across different cancer stage, age and gender subgroups. Even after adjusting for known confounders, unmarried patients were at greater risk of cancer-specific mortality. Social support aimed at this population could improve the likelihood of achieving cure.

It has been shown that married individuals have longer overall and cancer-specific survival (CSS) than those people who are single, widowed or divorced^1^,^2^. People who are married receive better social support, which subsequently promote health and survival^3^. Spouses can not only provide basic emotional support, but also facilitate the patients to receive more critical health care services^4^. Aizer *et al*. used the Surveillance, Epidemiology and End Results (SEER) database to study nearly 1 million contemporary cancer patients in the United States and found that unmarried patients, compared with married patients, are at higher risk of presentation with metastatic cancer, under-treatment, and death resulting from their corresponding cancer^5^. Thus, marital status is considered as an independent prognostic factor of survival in many cancers^5^,^6^,^7^,^8^. Prior investigations have also demonstrated that marital status plays a mixed or nonsignificant effect on disease-specific survival^9^,^10^,^11^. However, the role of marital status in affecting survival of patients with hepatocellular carcinoma (HCC) has not yet been assessed.

Liver cancer (LC) ranks the fifth most common malignancy and the third leading cause of cancer-related deaths globally^12^. HCC is the most common type of LC accounting for approximately 80 percent of all liver cancers^13^. We noticed that most studies only compare prognosis between married and unmarried individuals, and those separated, divorced and widowed patients were ignored without differentiating^5^. Given that 51 percent of Americans are married and HCC is one of the most common malignancies, targeted social support interventions could significantly prolong survival^5^,^14^. In this study, we searched the SEER population-based database of individuals diagnosed between 1988 and 2007 to evaluate discrepancies in survival trends among different marital status. Our primary objectives were to make generalizable conclusions regarding the survival discrepancies that might exist in these groups.

## Materials and Methods

### Patients

The SEER Cancer Statistics Review (http://seer.cancer.gov/data/citation.html), a report on the most recent cancer incidence, mortality, survival, prevalence, and lifetime risk statistics, is published annually by the Data Analysis and Interpretation Branch of the National Cancer Institute (Bethesda, MD). The current SEER database consists of 18 population-based cancer registries that represent approximately 26% of the population in the United States. SEER data contain no identifiers and are publicly available for studies of cancer-based epidemiology and survival analysis.

Cases of invasive HCC diagnosed between January 1, 1988, and December 31, 2007, were extracted from the SEER database (SEER*Stat 8.2.1) according to the Site Recode Classifications. Only those patients who underwent surgery at an age of between 18 and 85 years at diagnosis were included. Patients were excluded if they had incomplete staging, distant metastasis (M1), no evaluation of histological type, or follow-up. Age, sex, race, histologic type, stage, tumor grade, tumor size, and cancer-specific survival (CSS) rates were assessed. Adjuvant chemotherapy was not evaluated because the SEER registry does not include this information. The primary end point of the study is 5-year CSS rate, which was calculated from the date of diagnosis to the date of cancer-specific death. Cancer-specific deaths were treated as events, and deaths from other causes were treated as censored observations. The median follow-up period of patients was calculated from the date of diagnosis to the date of cancer-specific death. Marital status is coded as married, divorced, widowed, separated, and never married. Individuals in the separated and divorced group were clustered together as the divorced/separated group in this study.

This study was based on public data from the SEER database; we obtained permission to access research data files with the reference number 10504-Nov 2014. The data did not include the use of human subjects or personal identifying information. Thus, no informed consent was required for this part of the study.

### Statistical Analyses

Categorical variables were presented as frequency (%), and continuous variables were presented as median (interquartile range) or mean ± SD. The association between marital status categories and clinicopathological parameters was assessed using the chi-square (χ2) test. Continuous variables were compared using the Student t test. Survival curves were generated using the Kaplan-Meier method; differences between the curves were analyzed by using the log-rank test. Multivariable Cox proportional hazards regression models were used to assess potential risk factors for survival outcomes. All statistical analyses were performed using the statistical software package SPSS for Windows, version 17 (SPSS, Inc). The results were considered statistically significant when a 2-tailed test provided a P value of less than 0.05.

## Results

### Patient Characteristics

We identified 8621 eligible patients with HCC in the SEER database during the 20-year study period (between 1988 and 2007). A total of 6341 (73.6%) were men, and 2280 (26.4%) were women. Of these, 5457 (63.5%) were married, 1399 (16.2%) had never married, 940 (10.9%) were divorced/separated, and 825 (9.6%) were widowed. Patients who were widowed were less likely to be younger than 45 (0.5%), have less <3 cm tumor (8.5%) (P < 0.001). The rate of surgery performed was comparable between the married and widowed groups (84.4% vs 83.9%). Married patients were also less likely to present with advanced tumor and stage than widowed patients (P < 0.001). Patient demographics and pathologic features are summarized in Table 1.

### Clinicopathological Differences Between the Groups

As illustrated in Table 1, there were significant differences observed between the 4 groups, including the calendar years of diagnosis (more frequent in 2002–2007, 67.9%; P < 0.001), sex (more frequent in men, 73.6%; P < 0.001), age (more frequent in 45–60 and 61–75 years, 78.9%; P < 0.001), race (more frequent in Caucasian, 63.7%; P < 0.001), pathologic grade (less poor/undifferentiated in grade, 10.0%; P < 0.001), stage (more localized, 42.7%; P < 0.001), and tumor size (more >5 cm, 34.0%; P < 0.001).

### Impact of Marital Status on Survival Outcomes

**Figure 1:**
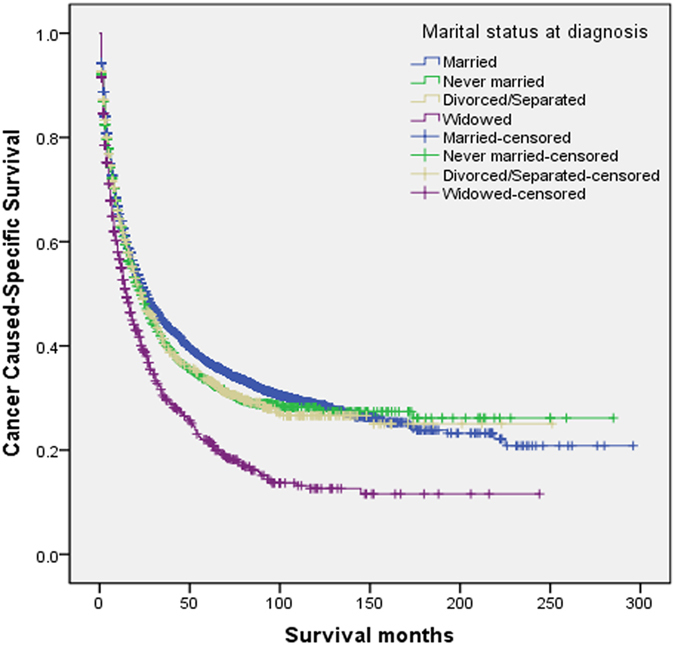
Survival curves in hepatocellular carcinoma patients according to marital status, χ2 = 77.744, P < 0.001.

The univariate log-rank test showed that the 3-year and 5-year CSS were 44.5% and 36.9% in the married group, 40.6% and 33.4% in the never married group, 40.2% and 33.5% in the divorced/separated group, 20.2% and 21.8% in the widowed group, respectively (*P* < 0.001) (Fig. 1). Moreover, an early year of diagnosis (1988–1994), men, age more than 75 years, African American race, poor/undifferentiated tumor grade, higher stage, and larger tumor size (*P* < 0.001) were regarded as significant risk factors by univariate analysis (Table 2). Multivariate analysis with Cox regression was performed, and the following 7 factors were found to be independent prognostic factors (Table 3), including year of diagnosis (1995–2001: HR, 0.974; 95% CI, 0.875–1.084; 2002–2007: HR, 0.803; 95% CI, 0.725–0.889), age (45–60 year: HR, 1.617; 95% CI, 1.441–1.814; 45–60 year: HR, 1.617; 95% CI, 1.441–1.814; 61–75 year: HR, 2.098; 95% CI, 1.869–2.355;>75 year: HR, 2.410; 95% CI, 2.115–2.747), race (African American: HR, 1.189; 95% CI, 1.092–1.295), pathological grading (poor/undifferentiated: HR, 1.396; 95% CI, 1.315–1.483), stage (regional: HR, 1.797; 95% CI, 1.694–1.907; distant: HR, 2.924; 95% CI, 2.692–3.177), tumor size (3–5 cm: HR, 1.736, 95% CI, 1.586–1.901; >5 cm: HR 2.529, 95% CI, 2.322–2.754), and marital status(never married: HR, 1.109, 95% CI, 1.025–1.200; divorced/separated: HR, 1.181, 95% CI, 1.082–1.288;. widowed: HR, 1.198, 95% CI, 1.093–1.312).

### Stratified Analysis of Marital Status Effect on CSS Rates

**Figure 2:**
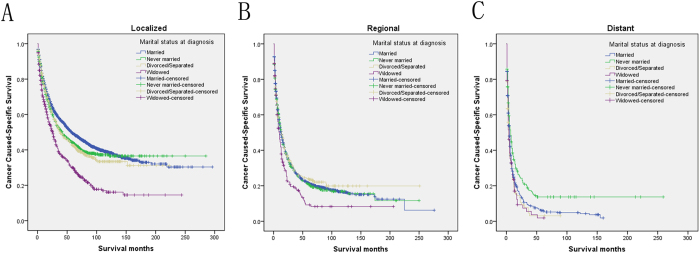
Subgroup analysis for evaluating the effect of marital status for hepatocellular carcinoma patients according different cancer stage. (**A**) The localized stage group: χ2 = 88.888, P < 0.001; (**B**) The regional stage group: χ2 = 12.846, P = 0.005; (**C**) The distant stage group: χ2 = 18.761, P < 0.001.

**Figure 3:**
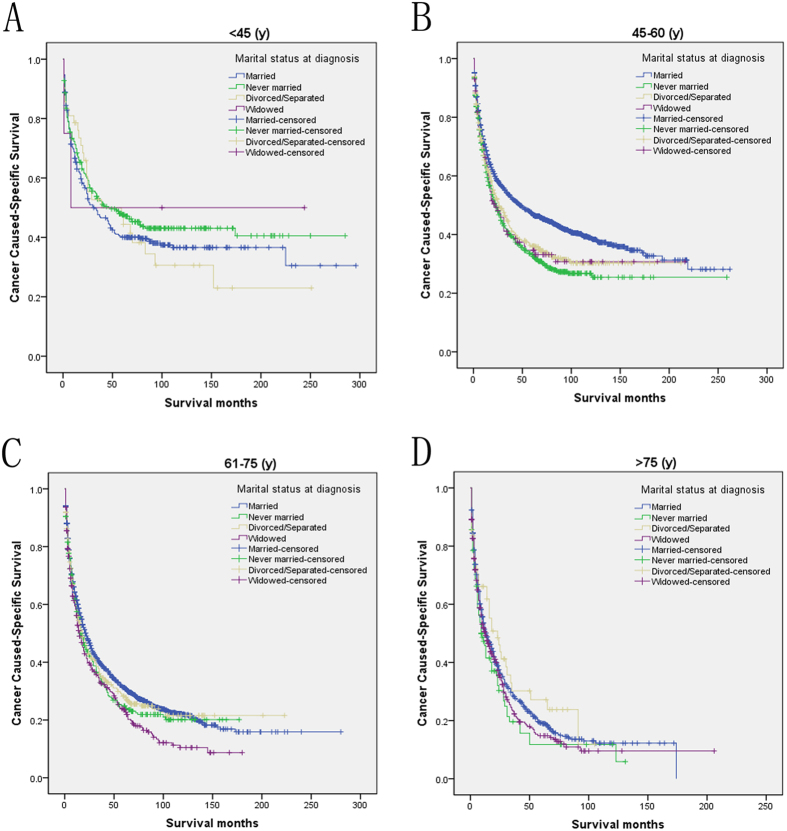
Subgroup analysis for evaluating the effect of marital status for hepatocellular carcinoma patients according different age. (**A**) <45 year: χ2 = 2.097, P = 0.553; (**B**) 45–60 year: χ2 = 46.729, P < 0.001; (**C**) 61–75 year: χ2 = 14.877, P = 0.002; (**D**) >75 year: χ2 = 5.327, P = 0.149.

**Figure 4:**
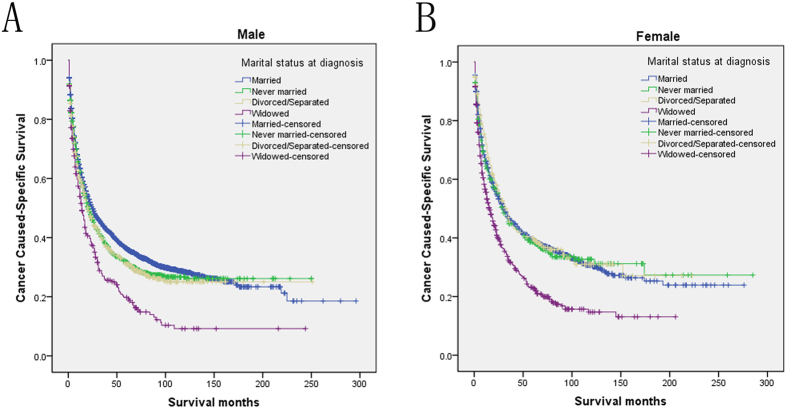
Subgroup analysis for evaluating the effect of marital status for hepatocellular carcinoma patients according different gender. (**A**) Male: χ2 = 43.265, P < 0.001; (**B**). Female: χ2 = 53.101, P < 0.001.

We then further analyzed the effect of marital status on CSS rates in each stage (Fig. 2). Both univariate and multivariate analysis showed that marital status was an independent prognostic factor in each tumor stage (*P* < 0.001). In addition to this, we also observed two interesting findings. First, the widowed group, compared with the other groups, always had the lowest CSS rate in the localized and regional stage. Widowed patients had 19.4% reduction in 5-year CSS compared with married patients in the localized stage (49.2% versus 29.8%, *P* < 0.001), 13.2% reduction in the regional stage (21.5% versus 8.3%, *P* < 0.001), 4.2% reduction in the distant stage (6.1% versus 1.9%, *P* = 0.166). Second, the divorced/separated group also had decreased 5-year CSS across several subgroups compared with patients in the never married group (Table 4). Furthermore, we made further stratified analysis of survival rates and hazard by gender and age (Figs 3 and 4). Unmarried patients always had the lowest CSS rate, which were consistent with aboved results (Table 5 and 6).

## Discussion

Despite the impact of marriage on cancer survival has been performed in some studies^15^,^16^,^17^, no research has been focused on the heterogeneity of unmarried patients in HCC or performed on stage by stage comparisons of the impact of marital status on survival. Our study showed that unmarried patients, including the widowed ones, are at significantly greater risk of death resulting from their cancer when compared with married patients. This survival discrepancy existed in each stage, age and gender. In addition, after adjusting for sex, pathological grading, stage, etc., marital status remained to serve as an independent prognostic predictor. Meanwhile, we also obeserved that more cancer cases were diagnosed in later years (more frequent in 2002–2007) which could be atrributed to the inclusion of more cancer registries in the SEER database over the years.

Being married has been shown to possess a survival disadvantage for patients with many types of cancers^18^,^19^. Delayed diagnosis and under-treatment are the mainly reported reasons of poor survival in unmarried patients^5^,^20^. In our study, we found that the percentage of patients with HCC in the widowed group (63.2%) was the highest in the localized stage compare with married (60.7%), never married (57.2%), and divorced/separated group (62.2%). Apparently, delayed diagnosis could not explain the result because the widowed group had the highest percentage. Another reason can be explain the unfavorable prognosis of unmarried individuals was under-treatment. However, surgery, rather than adjunctive therapy, is recommended for those resectable HCC patients. Interestingly, we found that the widowed patients, compared with those in the married group, still had a disadvantage of 19.4% in the localized stage, 13.2% in the regional stage and 4.2% in the distant stage regarding the 5-year CSS. Unmarried patients were at an increased risk of cancer mortality in contrast to married patients with different gender and age subgroups after adjusted for confounding factors. When comparing with married patients, widowed patients always had the worse CSS in all subgroups. Besides, no significantly difference of surgical resection rates was observed between the married and widowed groups. Thus, the hypothesis of under-treatment could not be supported by these findings.

Married patients have better adherence with prescribed treatments than unmarried patients. Delayed radiation treatments in head/neck cancer patients due to impaired adherence can result in increased rates of recurrence and poorer survival^21^. Similar results are also observed in other cancers^22^,^23^. Support systems, ranging from financial to emotional, are always lacking in unmarried patients. Spouses can provide adequate financial support to cover the costs of cancer treatment. Contrarily, unmarried patients might be reluctant to receive the treatment they needed due to economic reasons. Other than financial support, patients also have an emotion pillar to lean on provided by spouses during some of the more difficult times of their lives. Schlegel et al, also demonstrated that single patients had higher rates of depression^24^.

Psychologically, unmarried patients display more stress and depression when they are diagnosed with cancer, which can alter immune function and result in tumor progression^25^,^26^. DiMatteo *et al*. reported that married patients displayed lower risk of depression^27^. Moreover, Goodwin *et al*. found that women with depression were at greater risk for undergoing non-definitive treatment and display worse survival after a diagnosis of breast cancer^28^. A perceived lack of social support was associated with higher cortisol levels in patients with cancer, and chronic stress might promote cortisol secretion^29^,^30^. Lower natural-killer cell count and survival was also observed in those patients whom lack of social support^31^. Increased cortisol levels may downregulate the cortisol receptors, thus reduce anti-inflammatory response and promote inflammation^32^. In addition, a five year observational cohort study demonstrated that depression and anxiety were correlated with breast cancer recurrence^33^. Stress mediators produced in chronic stress could result in tumor metastasis through activation of specific signaling pathways and the tumor microenvironment^25^.

Although this study is based on a large population and partly answer the questions about marital status and prognosis in HCC, potential limitations should also be considered. First, the SEER database only collects the marital status at diagnosis, which could serve as a time dependent variable and may be changed after diagnosis. The changed marital status could also affect survival. Second, the information on smoking and alcohol use may not be available in SEER, and some studies have reported that unmarried patients may be at greater risk of such habits^34^. Furthermore, the SEER database also lacks important information regarding therapy options, income/insurance status, education and quality of marriage, which could not be adjusted by our analyses. Importantly, due to the retrospective nature, psychological tests could not be used to validate our hypothesis that psychosocial factors may be the main reasons for poor survival in unmarried patients.

Despite these limitations, our study indicates that unmarried patients are at greater risk of delayed diagnosis and cancer-specific mortality. Our study also reveals that unmarried patients groups form essentially a heterogeneous group, and widowed patients are always at the highest risk of mortality. Physicians caring for unmarried patients with HCC, especially in widowed ones, should realize the poorer outcomes in this population. It raises the possibility that investments in targeted social support services and interventions aimed at this population could significantly improve the likelihood of achieving cure.

## Additional Information

**How to cite this article:** Zhang, W. *et al*. Prognostic value of marital status on stage at diagnosis in hepatocellular carcinoma. *Sci. Rep.* **7**, 41695; doi: 10.1038/srep41695 (2017).

**Publisher's note:** Springer Nature remains neutral with regard to jurisdictional claims in published maps and institutional affiliations.

## Figures and Tables

**Table 1:** Characteristics of Patients from SEER Database by marital status.

Characteristic	No. (%) of patients					
	Total	Married	Never married	Divorced/ Separated	Widowed	P value
	n = 8621	n = 5457	n = 1399	n = 940	n = 825	
Media follow up (mo)	37	40	36	36	25	
(IQR)	5–63	6–67	5–60	5–62	4–31	
Years of diagnosis						P < 0.001
1988–1994	557(6.5)	404(7.4)	51(3.6)	39(4.1)	63(7.6)	
1995–2001	2212(25.6)	1450(26.6)	319(22.8)	192(20.4)	251(30.4)	
2002–2007	5852(67.9)	3603(66.0)	1029(73.6)	709(75.5)	511(62.0)	
Sex						P < 0.001
Male	6341(73.6)	4306(78.9)	1058(75.6)	688(73.2)	289(35.0)	
Female	2280(26.4)	1151(21.1)	341(24.4)	252(26.8)	536(65.0)	
Age						P < 0.001
<45	659(7.6)	322(5.9)	291(20,8)	42(4.5)	4(0.5)	
45–60	3520(40.8)	2199(40.3)	701(50.1)	520(55.3)	100(12.1)	
61–75	3283(38.1)	2276(41.7)	323(23.1)	323(34.4)	361(43.8)	
>75	1159(13.5)	660(12.1)	84(6.0)	55(5.9)	360(43.6)	
Race						P < 0.001
Caucasian	5425(63.7)	3361(61.6)	878(62.8)	667(71.0)	519(62.9)	
African American	914(11.4)	384(7.0)	312(22.3)	137(14.6)	81(9.8)	
Others*	2282(24.9)	1712(31.4)	209(14.9)	136(14.4)	225(27.3)	
Pathological grading						0.677
High/Moderate	6564(28.3)	4147(76.0)	1055(75.4)	724(77.0)	638(77.3)	
Poor/UD	2057(10.0)	1310(24.0)	344(24.6)	216(23.0)	187(22.7)	
Stage						0.015
Localized	5216(42.7)	3310(60.7)	800(57.2)	584(62.2)	522(63.2)	
Regional	2471(27.2)	1584(29.0)	424(30.3)	257(27.3)	206(25.0)	
Distant	934(15.9)	563(10.3)	175(12.5)	99(10.5)	97(11.8)	
Tumor size						P < 0.001
<3 cm	1747(12.8)	1125(20.6)	267(19.1)	220(23.4)	135(16.4)	
3–5 cm	2434(19.8)	1513(27.7)	383(27.4)	319(33.9)	219(26.5)	
>5 cm	4440(34.0)	2819(51.7)	749(53.5)	401(42.7)	471(57.1)	

*Including other (American Indian/AK Native, Asian/Pacific Islander) and unknowns.

**Table 2:** Univariate survival analyses of HCC patients according to various clinicopathological variables.

Variable	n	3-year CSS (%)	5-year CSS (%)	Log rank χ2 test	P
Years of diagnosis				124.997	P < 0.001
1988–1994	557	29.7%	23.0%		
1995–2001	2212	35.4%	27.8%		
2002–2007	5852	45.8%	38.3%		
Sex				1.991	0.158
Male	6341	41.8%	34.3%		
Female	2280	42.9%	35.7%		
Age				309.794	P < 0.001
<45	659	50.2%	43.9%		
45–60	3520	49.1%	42.5%		
61–75	3283	37.9%	29.5%		
>75	1159	26.6%	17.7%		
Race				38.560	P < 0.001
Caucasian	5425	42.1%	35.1%		
African American	914	34.0%	26.0%		
Others*	2282	45.3%	36.9%		
Pathological grading				327.616	P < 0.001
High/Moderate	6564	45.6%	38.4%		
Poor/undifferentiation	2057	28.0%	22.4%		
Stage				1440.866	P < 0.001
Localized	5216	54.6%	45.9%		
Regional	2471	27.2%	20.7%		
Distant	934	10.1%	6.7%		
Tumor size (mm)				1019.417	P < 0.001
<3 cm	1747	69.9%	62.2%		
3–5 cm	2434	46.7%	38.5%		
>5 cm	4440	28.3%	21.3%		
Marital Status				77.744	P < 0.001
Married	5457	44.5%	36.9%		
Never married	1399	40.6%	33.4%		
Divorced/Separated	940	40.2%	33.5%		
Widowed	825	20.2%	21.8%		

*Including other (American Indian/AK Native, Asian/Pacific Islander) and unknowns.

**Table 3:** Multivariate Cox model analyses of prognostic factors of HCC.

Variable	Hazard Ratio	95% CI	P
Years of diagnosis			P < 0.001
1988–1994	1	Reference	
1995–2001	0.974	0.875–1.084	
2002–2007	0.803	0.725–0.889	
Age			P < 0.001
<45	1	Reference	
45–60	1.617	1.441–1.814	
61–75	2.098	1.869–2.355	
>75	2.410	2.115–2.747	
Race			P < 0.001
Caucasian	1	Reference	
African American	1.189	1.092–1.295	
Others*	0.866	0.813–0.921	
Pathological grading			P < 0.001
High/Moderate	1	Reference	
Poor/undifferentiation	1.396	1.315–1.483	
Stage			P < 0.001
Localized	1	Reference	
Regional	1.797	1.694–1.907	
Distant	2.924	2.692–3.177	
Tumor size (mm)			P < 0.001
<3 cm	1	Reference	
3–5 cm	1.736	1.586–1.901	
>5 cm	2.529	2.322–2.754	
Marital Status			P < 0.001
Married	1	Reference	
Never married	1.109	1.025–1.200	
Divorced/Separated	1.181	1.082–1.288	
Widowed	1.198	1.093–1.312	

*Including other (American Indian/AK Native, Asian/Pacific Islander) and unknowns.

**Table 4:** Univariate and multivariate analyses for evaluating marital status influencing CSS in HCC based on different cancer stage.

Variable	5-year CSS (%)	Univariate analysis		Multivariate analysis	
		Log rank χ2 test	P	HR(95% CI)	P
Localized					
Marital status		88.888	P < 0.001		P < 0.001
Married	49.2%			Reference	
Never married	44.1%			1.259(1.128–1.404)	P < 0.001
Divorced/Separated	42.4%			1.265(1.123–1.424)	P < 0.001
Widowed	29.8%			1.264(1.121–1.425)	P < 0.001
Regional					
Marital status		12.846	0.005		0.045
Married	21.5%			Reference	
Never married	20.8%			1.089(0.953–1.243)	0.251
Divorced/Separated	23.5%			1.096(0.937–1.281)	0.010
Widowed	8.3%			1.265(1.057–1.514)	P < 0.001
Distant					
Marital status		18.761	P < 0.001		0.039
Married	6.1%			Reference	
Never married	13.7%			0.827(0.670–1.022)	0.079
Divorced/Separated	3.1%			1.192(0.942–1.509)	0.143
Widowed	1.9%			1.197(0.928–1.543)	0.166

**Table 5:** Univariate and multivariate analyses for evaluating marital status influencing CSS in HCC based on different age.

Variable	5-year CSS (%)	Univariate analysis		Multivariate analysis	
		Log rank χ2 test	P	HR(95%CI)	P
<45					
Marital status		2.097	0.553		NI
Married	40.0%				
Never married	47.6%				
Divorced/Separated	44.4%				
Widowed	NI				
45–60					
Marital status		46.729	P < 0.001		P < 0.001
Married	47.1%			Reference	
Never married	33.2%			1.377(1.231–1.541)	P < 0.001
Divorced/Separated	35.8%			1.347(1.189–1.525)	P < 0.001
Widowed	33.1%			1.852(1.430–2.397)	P < 0.001
61–75					
Marital status		14.877	0.002		0.001
Married	31.2%			Reference	
Never married	24.8%			1.057(0.916–1.219)	0.449
Divorced/Separated	27.8%			1.112(0.964–1.282)	0.146
Widowed	23.5%			1.320(1.154–1.510)	P < 0.001
>75					
Marital status		5.327	0.149		NI
Married	19.2%				
Never married	11.8%				
Divorced/Separated	23.8%				
Widowed	14.8%				

**Table 6:** Univariate and multivariate analyses for evaluating marital status influencing CSS in HCC based on different gender.

Variable	5-year CSS (%)	Univariate analysis		Multivariate analysis	
		Log rank χ2 test	P	HR(95%CI)	P
Male					
Marital status		43.265	P < 0.001		P < 0.001
Married	36.3%			Reference	
Never married	31.6%			1.141(1.043–1.247)	0.004
Divorced/Separated	31.0%			1.256(1.136–1.390)	P < 0.001
Widowed	19.2%			1.318(1.145–1.517)	P < 0.001
Female					
Marital status		53.101	P < 0.001		0.074
Married	39.3%			Reference	
Never married	38.2%			1.048(0.887–1.238)	0.580
Divorced/Separated	39.5%			1.040(0.872–1.240)	0.666
Widowed	22.9%			1.199(1.048–1.373)	0.008

NI: not included in multivariate survival analysis.

P values were adjusted for years of diagnosis, sex, age, race, pathological grading, stage and tumor size as covariates between the two groups.
